# Suspected Tumor-Related Hemorrhage as a Rare Complication of Stereotactic Body Radiotherapy in a Dog with Cranial Mediastinal Mass: A Case Report

**DOI:** 10.3390/vetsci12100982

**Published:** 2025-10-13

**Authors:** Jaewon Kim, Inseong Jeong, Chul Park, Younghwan Kim, Kidong Eom, Jaehwan Kim

**Affiliations:** 1 Department of Veterinary Medical Imaging, College of Veterinary Medicine, Konkuk University, Seoul 05029, Republic of Korea; solid0209@konkuk.ac.kr (J.K.);; 2 Royal Animal Medical Center, Seoul 02140, Republic of Korea

**Keywords:** dog, mediastinal mass, stereotactic radiotherapy, tumor-related hemorrhage

## Abstract

**Simple Summary:**

Stereotactic body radiotherapy is a highly targeted radiation treatment that has been used in dogs to manage mediastinal tumors such as thymomas and heart base tumors. Most side effects are mild and temporary, such as tiredness, radiation-induced inflammation of the lungs or esophagus, or abnormal heart rhythms. Although rare, bleeding within a tumor has been reported in people shortly after stereotactic body radiation therapy. Until now, such an acute hemorrhagic complication had not been documented in veterinary patients. This report describes a Pomeranian dog with a tumor in the cranial mediastinum that received stereotactic body radiation therapy. The day after the final treatment, the dog developed breathing difficulty and anemia. imaging and blood test results supported a diagnosis of tumor-associated bleeding. The patient recovered with supportive care and remained clinically stable during follow-up. In addition to presenting this rare complication, this case also discusses possible radiobiological mechanisms. Although rare, this type of hemorrhage can be life-threatening and may cause different symptoms depending on its location. Clinicians should be aware of this risk when planning treatment and managing patients.

**Abstract:**

Stereotactic body radiotherapy (SBRT) has been increasingly used in dogs for mediastinal tumors and is generally considered a precise and relatively safe treatment, with clinically significant complications reported only rarely. A cranial mediastinal mass was incidentally identified in a 10-year-old Pomeranian dog and cytologically diagnosed as a carcinoma. SBRT was performed using volumetric-modulated arc therapy, with a total dose of 27 Gy delivered in three fractions on alternate days. One day after completing treatment, the dog developed acute dyspnea and anemia. Thoracic radiography revealed mediastinal widening and pleural effusion. Subsequent imaging and hematological assessments suggested intra-tumoral hemorrhage and hematoma formation. The patient was managed conservatively with supportive therapy, resulting in gradual clinical improvement. Follow-up computed tomography (CT) demonstrated a 25% reduction in contrast-enhancing tumor volume, accompanied by a large non-enhancing region presumed to represent hematoma. Despite these changes, the patient remained clinically stable during follow-up. This case represents the first documented report of an acute hemorrhagic complication following SBRT in a veterinary patient, emphasizing the importance of awareness of this rare adverse event during treatment planning and client communication.

## 1. Introduction

In dogs, neoplasms of the cranial mediastinum—including thymomas, lymphomas, and neuroendocrine carcinomas such as ectopic thyroid carcinomas—may remain asymptomatic and are often discovered incidentally during routine examinations [1,2,3,4]. When clinical signs are present, they are typically nonspecific—vomiting, regurgitation, anorexia, cough, dyspnea, tachypnea, and lethargy—and may also be location-dependent, such as edema of the face and neck due to cranial vena cava compression or invasion (precaval syndrome) [2,3,4]. These masses are increasingly treated using stereotactic body radiotherapy (SBRT), including intensity-modulated radiation therapy and volumetric-modulated arc therapy (VMAT), due to their ability to deliver high radiation doses per fraction with precision to anatomically complex regions [5,6,7,8].

SBRT has been used in dogs to treat mediastinal tumors such as thymomas and heart base tumors, with reported adverse effects most commonly including radiation-induced pneumonitis, arrhythmia, esophagitis, and transient lethargy or anorexia. Importantly, across published clinical series, grade 3 or higher toxicities have been very rare, indicating that clinically significant complications of SBRT in canine mediastinal tumors occur only infrequently [5,6,7,8].

Tumor-related hemorrhage, although uncommon, is a recognized complication of SBRT in humans and is reported most frequently in intracranial disease, particularly during treatment of brain metastases [9,10,11]. Nevertheless, tumor-related hemorrhage has also been reported after SBRT to extracranial lesions [12]. Acute intratumoral bleed occurring within 3 h of the first fraction in a patient with primary renal cell carcinoma [12]. However, to the best of our knowledge, acute tumor-related hemorrhage following SBRT has not been previously reported in veterinary patients.

This case report describes a suspected acute hemorrhagic complication following VMAT-based SBRT, highlighting the clinical and imaging findings and discussing potential mechanisms and implications for future treatment planning.

## 2. Case Presentation

A 10-year-old intact male Pomeranian dog was incidentally found to have a cranial mediastinal mass during a routine checkup. No relevant past medical conditions were reported. According to the owner, there was no known family history or genetic predisposition. The dog lived indoors as a companion animal and exhibited no behavioral abnormalities or notable psychosocial stressors. Physical examination revealed a body weight of 6.4 kg, with no abnormalities detected in vital parameters or thoracic auscultation. Thoracic radiographs demonstrated a round, soft tissue opacity mass within the cranial mediastinum. Complete blood count (CBC) results were within normal limits, with platelet count (262 × 10^3^/μL; reference range: 200–500 × 10^3^/μL), red blood cell count (7.51 × 10^6^/μL; reference range: 5.5–8.5 × 10^6^/μL), hemoglobin (16.6 g/dL; reference: 12–18 g/dL) and hematocrit (49.7%; reference: 37–55%). Pre-contrast thoracic computed tomography (CT) revealed a round, soft tissue-attenuating mass (length: 34.3 mm; width: 35.6 mm; height: 31.2 mm) located within the cranial mediastinum. Intravenous administration of iodinated contrast medium revealed that the mass demonstrated heterogeneous contrast enhancement. The lesion was immediately adjacent to the cranial vena cava, right atrium, and right ventricle; however, no evidence of invasion into adjacent cardiovascular or thoracic structures was observed, and no compressive effect on the vasculature was identified. Thoracic lymph nodes were within normal limits, and no evidence of metastatic disease was identified. Pre- and post-contrast CT evaluation revealed no abnormalities of the thyroid glands, which were normal in size and morphology. Incidental findings included benign prostatic hyperplasia consistent with the patient’s intact male status, as well as urinary bladder calculi and a small renal cortical cyst.

**Figure 1:**
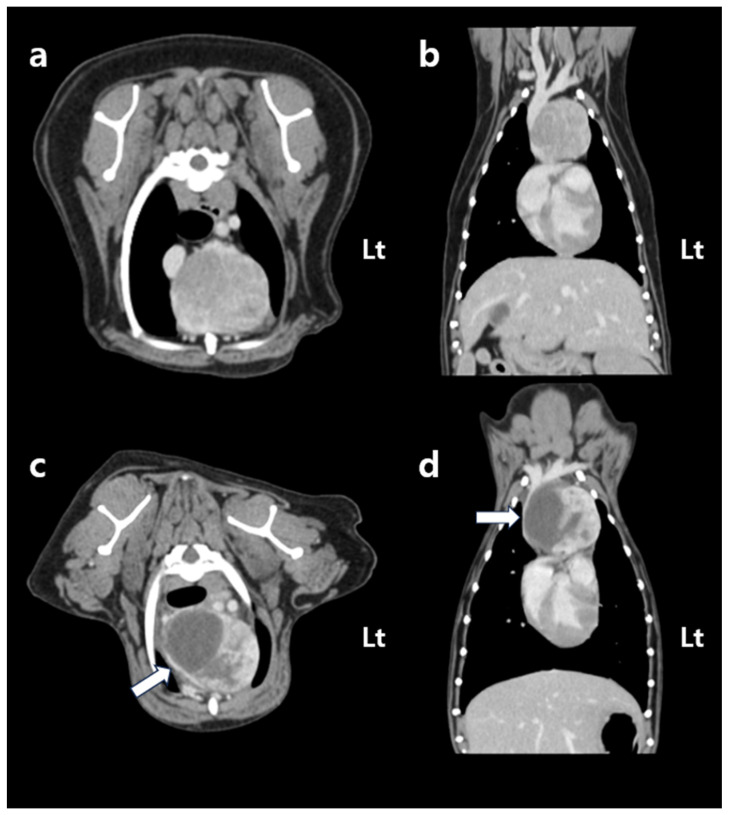
Post-contrast CT images acquired before (**a**,**b**) and 37 days after (**c**,**d**) the first fraction of stereotactic body radiotherapy. Transverse images are shown in (**a**,**c**), and dorsal reconstructions are shown in (**b**,**d**). All images were obtained using a soft tissue algorithm (window level, 45; window width, 450). Before stereotactic body radiotherapy, the cranial mediastinal mass displays heterogeneous contrast enhancement and is located in close proximity to the cranial vena cava, right atrium, and right ventricle. CT performed 37 days after the first radiation fraction shows that the overall size of the mass has increased; however, the volume of the contrast-enhancing region has decreased by approximately 25%. The cranial vena cava is compressed by a newly developed non-enhancing region (white arrow). CT, computed tomography. Lt: left.

Two weeks later, a CT scan was acquired for radiation therapy planning. The dog was positioned in ventral recumbency using a custom-made vacuum immobilization cushion (Chunsung, Seoul, Republic of Korea). Whole-body pre- and post-contrast CT images were obtained using a 64-multi-slice helical CT scanner (Optima CT660; GE HealthCare, Chicago, IL, USA) with the following parameters: 120 kVp, 160 mA, and 1.25 mm slice thickness. Intravenous contrast enhancement was achieved by administering 600 mg iodine/kg of iohexol (Omnipaque™ 300, GE HealthCare), using a power injector (MEDRAD^®^ Stellant, Bayer, Leverkusen, Germany). No significant changes in mass size, morphology, or contrast enhancement pattern were observed compared to the previous CT scan (Figure 1a,b).

At the time of radiation therapy planning, complete blood count results were within normal limits, with platelet count (302 × 10^3^/μL), red blood cell count (7.34 × 10^6^/μL), hemoglobin (16.7 g/dL), and hematocrit (52.4%). These findings indicated adequate hematologic status prior to initiation of SBRT.

**Figure 2:**
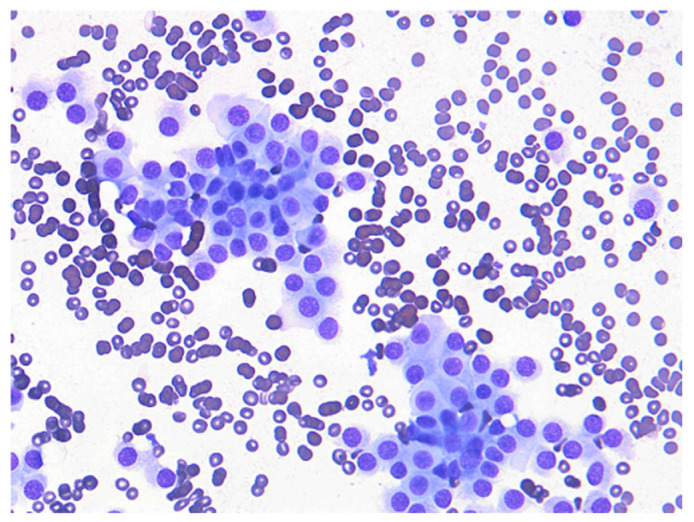
Cytologic features of the cranial mediastinal mass obtained by fine-needle aspiration (400×). Loosely cohesive clusters and individually scattered round cells are observed, with pale basophilic cytoplasm and eccentrically placed nuclei. Chromatin is finely to coarsely stippled with inconspicuous nucleoli. The cytomorphologic features are suggestive of carcinoma.

Ultrasound-guided fine-needle aspiration (FNA) of the mediastinal mass yielded cytologic findings consistent with a carcinoma. Cytology revealed loosely cohesive clusters of epithelial cells, with some groups displaying indistinct cytoplasmic borders, supporting a presumptive diagnosis of a neuroendocrine-type carcinoma. Given the anatomic location in the cranial mediastinum, immediately anterior to the right atrium and right ventricle, an ectopic thyroid carcinoma was regarded as the most plausible presumptive diagnosis (Figure 2). Other differential diagnoses, including chemodectoma and thymoma, were also considered. However, the cranial position of the mass, situated anterior to the heart base, reduced the likelihood of a chemodectoma. Moreover, thymoma was considered less likely owing to the absence of the abundant small lymphocyte population typically associated with thymic epithelial tumors, the lack of characteristic infiltration of well-differentiated mast cells on cytology, and the absence of overt squamous differentiation, which may be observed in thymic carcinoma. As ancillary diagnostic techniques such as immunocytochemistry and flow cytometry were not performed, the possibility of alternative diagnoses, including chemodectoma and thymoma, could not be entirely excluded.

**Figure 3:**
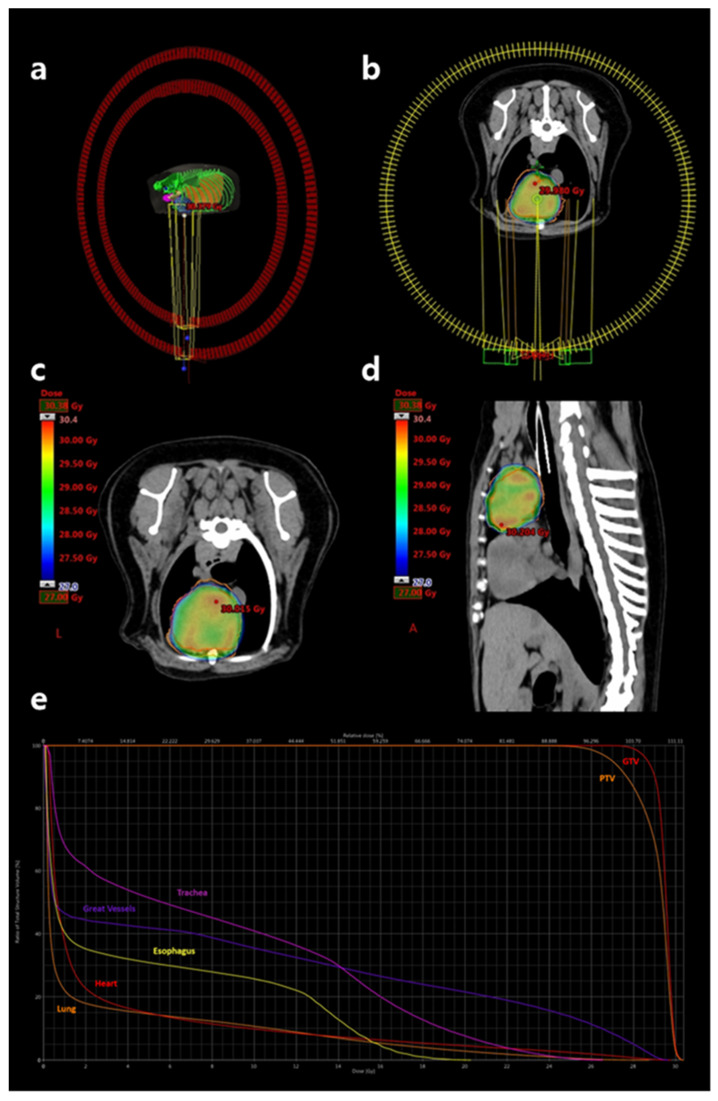
Radiation therapy planning performed using the Eclipse™ software version 13.7.33. (**a**,**b**) Schematic representations of the two coplanar 360-degree volumetric arcs used for stereotactic treatment. One arc rotates in a clockwise direction and the other in a counterclockwise direction. (**c**) Transverse and (**d**) sagittal views of the dose distribution over the cranial mediastinal mass. The dose color wash represents regions receiving doses equal to or greater than prescribed dose of 27 Gy, with the red dot indicating the maximum dose hotspot on the corresponding slice. (**e**) Dose–volume histogram illustrating target coverage and organ-at-risk sparing. From left to right: lung (orange), heart (red), esophagus (yellow), great vessels (purple), trachea (pink), PTV (orange), and GTV (red). The plan ensured that a minimum of 99% of the GTV and 95% of the PTV received at least 27 Gy. GTV, gross tumor volume; PTV, planning target volume.

SBRT was planned using VMAT and delivered via a 6 MV linear accelerator equipped with 5-mm multileaf collimators (Clinac iX; Varian Medical Systems, Palo Alto, CA, USA). Inverse treatment planning was performed using Eclipse™ software version 13.7.33 (Varian Medical Systems). A total dose of 27 Gy was prescribed in three fractions of 9 Gy, administered on alternate days. Two full-arc coplanar VMAT beams were used to optimize target coverage and dose conformity (Figure 3a,b).

The gross tumor volume (GTV) was defined as the contrast-enhancing portion of the cranial mediastinal mass. The planning target volume (PTV) was generated by applying a 3-mm isotropic expansion to the GTV to account for setup uncertainties and organ motion. Portions of the PTV overlapping sensitive organs at risk (OARs), including the heart, lungs, major vessels, and trachea, were cropped to limit dose delivery to these structures. The clinical target volume (CTV) was not defined. Dose planning ensured that 100% of the prescribed dose encompassed at least 95% of the PTV and 99% of the GTV. Organs at risk—including lungs, heart, esophagus, trachea, major vessels, bronchi, and spinal cord—were contoured and evaluated to meet appropriate dose constraints (Figure 3c–e). Dose constraints were established with reference to published human SBRT guidelines for three-fraction regimens [13,14,15]. Supplementary Table S1 summarizes the OAR dose constraints applied. The volumes of target structures and calculated radiation doses/volumes to those structures are summarized in Table 1.

Prior to each treatment fraction, a comprehensive clinical assessment was performed, including hematologic evaluation, measurement of body weight, and monitoring of vital parameters (temperature, pulse, and respiratory rate). Anesthesia was induced with alfaxalone and maintained with sevoflurane, with supplemental oxygen continuously administered throughout the procedure to ensure adequate systemic oxygenation.

**Figure 4:**
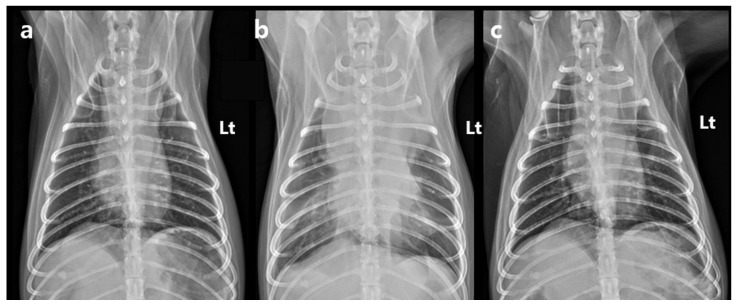
Serial ventrodorsal thoracic radiographs. (**a**) Thoracic radiograph obtained during radiation treatment planning, showing a mediastinal width of 44.7 mm. (**b**) Thoracic radiograph obtained 1 day after the final SBRT fraction (5 days after the first fraction of SBRT), demonstrating a marked increase in mediastinal width to 74.5 mm, increased soft tissue opacity within the mediastinum, and concurrent pleural effusion. (**c**) Thoracic radiograph obtained 92 days after the first fraction of SBRT, showing complete resolution of the pleural effusion and a reduction in mediastinal width to 43.4 mm. SBRT, stereotactic body radiotherapy. Lt: left.

**Figure 5:**
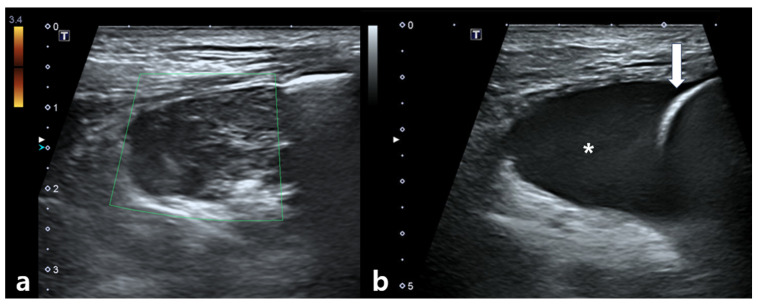
Thoracic ultrasonographic findings obtained 1 day after completion of the final fraction of stereotactic body radiotherapy. (**a**) A heterogeneous, hypoechoic mass can be detected adjacent to the irradiated mediastinal mass. No vascular flow is observed on color Doppler imaging. (**b**) Anechoic fluid accumulation is present between the lung parenchyma and thoracic wall, consistent with pleural effusion. The asterisk indicates fluid accumulation and the white arrow indicates the lung surface.

The dog remained clinically stable throughout the SBRT course. The dog completed all three planned SBRT fractions without treatment interruption and remained clinically stable under anesthesia during all sessions. However, the day after the final fraction (5 days after the first fraction), the dog developed acute dyspnea, characterized by increased inspiratory effort and mild open-mouth breathing. Clinical signs of anemia were also evident, as the mucous membranes appeared markedly pale, capillary refill time was prolonged to approximately 2.5 s, and the patient exhibited lethargy with reduced responsiveness to external stimuli. Hematologic testing revealed decreased platelet count (124 × 10^3^/μL), red blood cell count (4.04 × 10^6^/μL), hemoglobin (9.0 g/dL), and hematocrit (28.8%). To rule out an underlying coagulopathy, coagulation testing was performed, and the results showed that PT (10.4 s; reference range: 5–15 s), aPTT (29.2 s; reference range: 15–45 s), and fibrinogen concentration (2.7 g/L; reference range: 1–3 g/L) all remained within normal limits. Thoracic radiographs showed increased cranial mediastinal width (74.5 mm), swelling in submandibular and axillary regions, and pleural effusion (Figure 4). On palpation, the swelling in the bilateral submandibular and axillary regions was cool to the touch, soft, and pitting, with well-defined margins. There was no local warmth, erythema, or pain on palpation. The swelling was symmetrical and corresponded anatomically to the drainage area of the cranial vena cava, suggesting congestion secondary to venous compression rather than inflammatory or neoplastic infiltration. Thoracic ultrasonography revealed a heterogeneous, hypoechoic mass adjacent to the mediastinal lesion, with no detectable vascular flow signals on color Doppler imaging. Transthoracic echocardiography demonstrated a decreased left ventricular internal dimension during both diastole and systole, with preserved global systolic and diastolic function, and no evidence of structural cardiac abnormalities. Anechoic fluid accumulation was observed between the lung parenchyma and thoracic wall, consistent with pleural effusion (Figure 5). Based on the imaging and hematological findings, cranial vena cava compression secondary to tumor-related hemorrhage and hematoma formation associated with the irradiated mass was considered the most likely diagnosis. Other possible differential diagnoses included a necrotic portion of the tumor, abscess formation with inflammation, or a metastatic lesion.

The dog was hospitalized and managed with stabilization measures that included daily oxygen cage therapy, nebulization, and intravenous fluid therapy supplemented with vitamins B and C. Supportive medical treatment further consisted of vitamin K_1_ (0.1 mL/kg, SC, q8hr), tranexamic acid (10 mg/kg, IV, q8hr), Chlorpheniramine (0.2 mg/kg, IM, q12hr), and maintenance fluid therapy with normal saline.

**Figure 6:**
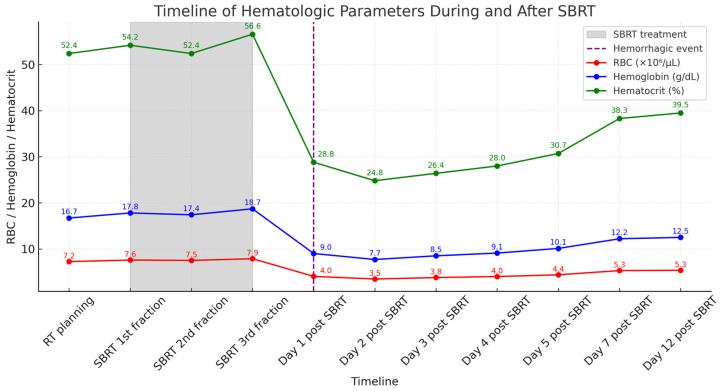
Timeline of hematologic parameters during and after stereotactic body radiotherapy (SBRT). Red blood cell count (RBC), hemoglobin concentration, and hematocrit values are plotted over time. The shaded area indicates the SBRT treatment period, which began 6 days after RT planning and was delivered every other day for a total of three fractions. A hemorrhagic event occurred on Day 1 post-SBRT, indicated by the dashed vertical line. The patient showed gradual hematologic recovery with supportive care and was discharged on Day 4 post-SBRT.

On day 2 post-SBRT (6 days after the first fraction of SBRT), anemia worsened with further decreases in the red blood cell count, hemoglobin concentration, and hematocrit to 3.46 × 10^6^/μL, 7.7 g/dL, and 24.8%, respectively. Thoracic radiography revealed a slight decrease in cranial mediastinal width (to 70.5 mm). Following conservative management with intravenous fluid therapy and supportive care, the blood results began to progressively improve. On day 3 post-SBRT (7 days after the first fraction of SBRT), red blood cell count increased to 3.78 × 10^6^/μL, hemoglobin concentration to 8.5 g/dL, and hematocrit to 26.4%. Thoracic radiography revealed a further small decrease in cranial mediastinal width (to 68.5 mm). By day 4 post-SBRT (8 days after the first fraction of SBRT), the red blood cell count, hemoglobin concentration, and hematocrit increased again to 4.00 × 10^6^/μL, 9.1 g/dL, and 28.0%, respectively. Mediastinal width decreased again to 63.7 mm, and soft tissue swelling in the submandibular and axillary regions was resolved, at which point the dog was discharged. Follow-up evaluations performed after discharge (on days 5, 7, and 12 post-SBRT) revealed that the patient remained clinically stable without recurrence of dyspnea. Hematologic parameters continued to improve, with red blood cell count, hemoglobin concentration, and hematocrit increasing to 4.38 × 10^6^/μL, 10.1 g/dL, and 30.7% on day 5 post-SBRT (9 days after the first fraction of SBRT); 5.29 × 10^6^/μL, 12.2 g/dL, and 38.3% on day 7 post-SBRT (11 days after the first fraction of SBRT); and 5.35 × 10^6^/μL, 12.5 g/dL, and 39.5% on day 12 post-SBRT (16 days after the first fraction of SBRT), respectively. Importantly, reticulocyte counts and percentages, which had remained within reference intervals (count: 8.4–129.3 × 10^9^/L; percentage: 0.1–2.0%) through day 4 post-SBRT, showed a marked increase during subsequent follow-up. On day 5 post-SBRT, reticulocyte values rose to 178.4 × 10^9^/L (4.07%), exceeding the upper reference limit. This regenerative response became more pronounced on day 7 post-SBRT, when reticulocytes peaked at 391.3 × 10^9^/L (7.4%). By day 12 post-SBRT, reticulocyte numbers remained elevated at 365.6 × 10^9^/L (6.84%). This temporal pattern—initially normal reticulocyte levels followed by a delayed and robust increase—provides clear evidence of a regenerative response, consistent with recovery from acute hemorrhagic anemia. The sequential changes in hematologic parameters are summarized according to the clinical timeline (Figure 6). Serial hematologic data are detailed in Supplementary Table S2.

At follow-up, the dog remained in a stable condition, with normal respiratory function and no recurrence of swelling in the submandibular or axillary region. Follow-up CT was performed on day 33 post-SBRT (37 days after the first fraction of SBRT), not primarily to assess tumor shrinkage but rather to evaluate potential treatment-related complications. Specifically, the scan was intended to monitor for tumor-associated hemorrhage and hematoma formation, to assess compression of adjacent vascular and mediastinal structures, and to rule out bleeding from surrounding normal organs. The CT showed an overall increase in tumor dimensions (length: 42.1 mm; width: 49.0 mm; height: 40.0 mm). Tumor volume increased from 25.0 cm^3^ to 46.4 cm^3^; however, the contrast-enhancing portion decreased by approximately 25% to 18.7 cm^3^. This decrease was accompanied by a relative increase in the non-enhancing component of the mass (pre-contrast: 30 HU; post-contrast: 31.5 HU), which exerted compression on the cranial vena cava (Figure 1c,d). Based on imaging features, hematologic findings, and the clinical course, the non-enhancing region was interpreted as a tumor-related hemorrhage secondary to radiation therapy. Thoracic radiography performed on day 88 post-SBRT (92 days after the first fraction of SBRT) revealed a further reduction in the mediastinal width to 43.4 mm, with no evidence of pleural effusion (Figure 4). The dog remained clinically stable, without any evidence of dyspnea, lethargy, or other signs of anemia. At the last follow-up on day 143 after the first SBRT fraction (139 days after completion of the final fraction), the patient was still clinically stable without recurrence of dyspnea or anemia-related signs. Thoracic radiographs at that time showed no significant changes compared with those obtained on day 88 post-SBRT (Figure 4c).

## 3. Discussion

Radiation therapy is widely used as a hemostatic treatment in human oncology for patients with advanced or incurable cancers complicated by clinically significant bleeding [16,17]. Reported indications include head and neck tumors (oral cavity, tongue, oropharynx, and hemorrhagic cervical lymph nodes), thoracic tumors such as lung cancer presenting with hemoptysis, gastrointestinal tumors including gastric, rectal, and esophageal cancers, genitourinary tumors such as bladder, prostate, cervical, endometrial, and ovarian cancers, and even cutaneous malignancies [16,17]. A variety of hypofractionated palliative regimens have been employed, most commonly 40 Gy in 15 fractions, 30 Gy in 10 fractions, 20 Gy in 5 fractions, 15 Gy in 5 fractions, or single-fraction treatments of 8 Gy or 4 Gy, depending on tumor location, bleeding severity, and patient performance status [16,17]. These protocols have achieved hemostatic response rates of approximately 70–90% across multiple tumor types [16].

In veterinary oncology, although evidence is limited, radiotherapy has also been reported to exert hemostatic effects in bleeding tumors. Notably, resolution of hemorrhage has been described in dogs with adrenal pheochromocytomas complicated by hemoperitoneum, where stereotactic protocols such as 33 Gy in 3 consecutive daily fractions or 35 Gy in 5 fractions delivered every other day achieved clinical stabilization and cessation of bleeding [18]. Similarly, in a case of inoperable hemorrhagic oral melanoma, palliative radiotherapy using 36 Gy in 6 weekly fractions successfully controlled persistent oral bleeding, improved hemoglobin levels, and alleviated clinical signs [19]. These reports illustrate that, as in human oncology, radiotherapy can provide effective hemostasis in selected veterinary patients, using both stereotactic and palliative fractionation regimens [18,19].

The hemostatic effect is attributed to radiation-induced vascular damage leading to endothelial injury, thrombosis, and ultimately fibrosis, thereby stabilizing or sealing the hemorrhagic vessels [16,17,18,19].

Despite the hemostatic effect, there are occasional reports of hemorrhagic complications following radiation therapy, particularly with high-dose stereotactic techniques [9,10,11,12]. Tumor-related hemorrhage following radiation therapy has been reported in human medicine, primarily in the context of brain metastases treated with stereotactic radiosurgery (SRS) [9,10,11]. However, this complication is rare. A systematic review determined that only 0.8% of patients with brain metastases who underwent SRS developed tumor hemorrhage within 72 h [9]. The affected patients tended to have brain metastases arising from highly vascular tumors such as renal cell carcinoma and lung cancer [9]. Another retrospective analysis reported symptomatic hemorrhage in approximately 5.6% of patients with brain metastases who underwent SRS, indicating that this adverse event is uncommon and typically unpredictable [10].

Although this complication has not previously been reported in the veterinary literature, the established radiobiological mechanisms underlying high-dose radiation therapy provide a theoretical basis to explain the occurrence of tumor-related hemorrhage following SBRT. High-dose hypofractionated radiotherapies—such as SBRT and SRS—exert tumoricidal effects through direct DNA damage and indirect mechanisms, such as vascular injury and subsequent disruption of the tumor microenvironment [20]. Studies have shown that radiation doses exceeding 10–15 Gy per fraction can induce endothelial cell apoptosis, increase vascular permeability, and compromise perfusion within tumors, leading to ischemia and secondary tumor cell death [20,21]. This reduces the survival of clonogenic tumor cells and creates areas of hemorrhagic necrosis, particularly in highly vascular tumors [20,21]. This can result in the breakdown of capillary walls, extravasation of blood components, and in some cases, formation of hematomas within or around the irradiated mass [20]. These hallmarks of radiation-induced indirect cytotoxicity can occur within days of treatment [20,21].

While the prescribed SBRT dose (27 Gy in 3 fractions) was slightly below thresholds typically associated with vascular injury in human studies, several biological factors may explain the occurrence of hemorrhage in this canine case. Importantly, tumor blood vessels are structurally and functionally abnormal [20]. The vessel walls often consist of a single layer of irregularly shaped endothelial cells, loosely connected with wide intercellular gaps that are sometimes filled by tumor cells [20]. These endothelial layers are supported by incomplete basement membranes and poorly organized pericytes, and they may even include cells derived from bone marrow-derived endothelial progenitors, transdifferentiated mesenchymal cells, or myeloid cells [20]. Such immature and disorganized vasculature is prone to irregular dilation, constriction, and branching, rendering it inherently fragile and highly susceptible to ionizing radiation [20]. This inherent fragility may reduce the effective dose threshold for vascular injury relative to normal vasculature. Taken together, these tumor-specific vascular characteristics provide a plausible explanation for why clinically significant hemorrhage occurred in this patient despite the prescribed dose being slightly below the thresholds commonly associated with vascular damage in human literature.

This case highlights tumor-related hemorrhage as a rare, but potentially life-threatening, complication of SBRT. Although there are limited reports of this adverse event in humans, primarily in association with intracranial metastases, studies describing this complication in veterinary medicine are lacking. To the best of our knowledge, this is the first report of suspected post-radiation hemorrhage in a canine mediastinal neuroendocrine carcinoma treated with VMAT-based SBRT. Given the proximity of thoracic tumors to critical vascular structures, such as the cranial vena cava, even a limited hemorrhagic event can result in substantial clinical consequences, including major vessel compression, impaired venous return, and respiratory compromise. Furthermore, the clinical presentation of radiation-associated hemorrhage may vary considerably depending on tumor location, making early diagnosis challenging. Therefore, awareness of this potential complication is essential when planning stereotactic radiotherapy for tumors located near major vasculature. Although the overall risk is low, appropriate monitoring strategies and client education should be considered, especially when SBRT is being administered.

Tumor lysis syndrome (TLS) was considered as a potential complication in this case, since it has been reported in dogs following radiotherapy for lymphoma and, more recently, for thymoma containing abundant lymphocytes [22,23]. TLS results from the rapid release of intracellular contents such as potassium, phosphorus, and nucleic acids, leading to characteristic metabolic derangements including hyperkalemia, hyperphosphatemia, hypocalcemia, metabolic acidosis, and acute kidney injury [22,23]. In dogs, TLS has most frequently been described in hematopoietic tumors, but it has also been reported in solid tumors [22]. In the present case, the biochemical profile did not fulfill the diagnostic criteria for TLS. Blood urea nitrogen (BUN) fluctuated within the reference interval and creatinine remained normal-to-low. Phosphorus showed a slight within-reference increase and C-reactive protein (CRP) rose markedly, with no concurrent hyperkalemia or hypocalcemia. The complete sequential values are provided in Supplementary Table S3. In addition, clinical signs commonly associated with TLS, such as renal failure, arrhythmia, seizures, or sudden death, were not observed [22,23]. Instead, the predominant clinical findings were acute anemia, dyspnea, and rapid mediastinal enlargement, which were more consistent with an acute hemorrhagic event. Taken together, TLS was considered unlikely to be the primary cause of the clinical deterioration in this patient. Nevertheless, given recent reports of RT-induced TLS in canine thymoma and lymphoma, the potential risk of TLS should still be recognized in dogs with large, mediastinal tumors undergoing radiotherapy, and careful biochemical monitoring is recommended [22,23].

Other potential causes of hemorrhage should also be considered in this case. Idiopathic thymic hemorrhage—most often described in dogs under 2 years of age—has been attributed to structural fragility of intrathymic vessels during thymic involution, with rupture potentially precipitated by excitement or exertion [24]. In contrast, beyond approximately 2 years of age, the thymus undergoes involution with sclerosis of thymic vessels, which reduces the propensity for rupture [24]. In view of our patient’s geriatric age and cytology favoring a non-thymic origin, this entity was considered unlikely. Spontaneous tumor bleeding has been reported in both human and veterinary oncology, independent of radiation exposure; however, the very close temporal association with SBRT in this patient makes this explanation less likely as the sole cause. Coagulopathy was another possible differential, but coagulation testing (PT, aPTT) and platelet counts were within normal limits, reducing the likelihood of an underlying clotting disorder. Structural vascular abnormalities or pre-existing vascular injury were not identified on the planning CT scan prior to treatment, making an anatomic predisposition less likely. Finally, radiation-induced injury to adjacent normal vessels was also considered; however, the recheck CT did not reveal vascular damage, and dose constraints for the great vessels and heart were within accepted limits. Although these alternative etiologies cannot be completely excluded in the absence of histopathological confirmation, the imaging findings and clinical course most strongly support a radiation-associated vascular injury of the tumor vasculature as the primary cause of hemorrhage in this patient.

Previous studies in human radiosurgery and stereotactic radiotherapy have identified several factors associated with an increased risk of tumor-related hemorrhage (TRH), including larger tumor size or volume, higher maximum point dose, use of a greater number of isocenters, female sex, and prior therapy with immune checkpoint inhibitors [9,10]. To mitigate this risk, fractionation strategies have been proposed, particularly lowering the per-fraction dose while increasing the number of fractions, which may reduce acute stress on fragile tumor vasculature and lessen the likelihood of acute hemorrhage [9]. Although the risk of acute hemorrhage is highest in single-fraction stereotactic radiosurgery due to the very high per-fraction dose, the present case demonstrates that clinically significant bleeding can also occur with hypofractionated regimens, such as 9 Gy delivered in three fractions. The risk of intratumoral hemorrhage may be influenced not only by the total prescribed dose but also by the fractionation scheme. Within SBRT protocols, regimens employing lower per-fraction doses with a greater number of fractions may theoretically reduce vascular stress and thereby lower the likelihood of hemorrhagic events compared with more aggressive hypofractionated schedules [9,10]. Nevertheless, the present case represents a single clinical observation, and definitive conclusions regarding the optimal fractionation strategy for minimizing acute hemorrhagic risk in veterinary SBRT cannot be drawn. Further investigation in larger cohorts is warranted to better define safe and effective protocols.

This report is inherently limited by its description of a single case, which precludes the drawing of definitive conclusions regarding the causality between SBRT and the observed hemorrhagic complication. A major limitation is the absence of histopathological confirmation of the hemorrhage, which restricts diagnostic certainty and prevents definitive exclusion of other potential etiologies such as coagulopathy or spontaneous tumor rupture unrelated to radiation. Another important limitation is the lack of interval imaging between the final SBRT fraction and the onset of clinical signs, which makes it impossible to accurately determine the exact timing and temporal progression of the hemorrhage. The cytologic diagnosis was based solely on fine-needle aspiration, without adjunctive immunocytochemistry or flow cytometry. While the findings most strongly suggested an ectopic thyroid carcinoma, other differential diagnoses, including chemodectoma and thymoma, could not be definitively excluded. While the temporal association and imaging findings strongly support a radiation-induced vascular event, these limitations should be acknowledged when interpreting the findings. Finally, although the dose and fractionation used in this case were slightly below the thresholds commonly associated with radiation-induced vascular damage, tumor-specific factors such as inherent vascular fragility may have contributed; this possibility warrants further investigation in larger cohorts.

Despite these limitations, this case highlights a rarely reported but clinically important potential complication of SBRT in veterinary medicine. This adverse event can be life-threatening and may present with diverse clinical manifestations depending on the anatomical location of the hemorrhage. Clinicians should be aware of this risk when performing radiation therapy planning.

## 4. Conclusions

This case highlights suspected acute tumor-related hemorrhage as a rare but potentially serious complication of stereotactic body radiation therapy in dogs. Although this adverse event has occasionally been reported in human patients, particularly those with brain metastases, it has not previously been documented in veterinary medicine. The underlying mechanism may involve radiation-induced vascular injury and hemorrhagic necrosis, especially in tumors with fragile vasculature. While histological confirmation was not available in this case, the temporal association and imaging findings suggest a causal link. Clinicians should consider this possibility during treatment planning and monitor patients carefully when administering high-dose radiation therapy.

## Figures and Tables

**Table 1:** Summary of dose and volume data.

	Volume	Dmax	Dmean	Dmin	D2%	D50%	D98%
GTV	25 cm^3^	30.37 Gy	29.47 Gy	26.56 Gy	30.06 Gy	29.54 Gy	28.26 Gy
PTV	38.3 cm^3^	30.37 Gy	29.05 Gy	22.50 Gy	30.03 Gy	29.38 Gy	26.25 Gy
Heart	64 cm^3^	29.79 Gy	3.00 Gy	0.18 Gy	25.84 Gy	0.65 Gy	0.21 Gy
Lung	302.7 cm^3^	28.73 Gy	2.45 Gy	0.05 Gy	21.68 Gy	0.24 Gy	0.08 Gy
Great vessels	11.6 cm^3^	29.65 Gy	8.33 Gy	0.01 Gy	28.77 Gy	0.53 Gy	0.04 Gy
Trachea	14.9 cm^3^	26.57 Gy	8.09 Gy	0.13 Gy	23.38 Gy	5.62 Gy	0.25 Gy
Esophagus	7.0 cm^3^	20.29 Gy	4.43 Gy	0.09 Gy	17.17 Gy	0.55 Gy	0.10 Gy
Spinal cord	7.1 cm^3^	9.52 Gy	2.27 Gy	0.01 Gy	8.83 Gy	0.39 Gy	0.03 Gy

## Data Availability

The original contributions presented in this study are included in the article/Supplementary Material. Further inquiries can be directed to the corresponding author.

## Supplementary Materials

The following supporting information can be downloaded at: https://www.mdpi.com/article/10.3390/vetsci12100982/s1, Table S1: Organ-at-risk (OAR) dose constraints applied (3-fraction SBRT protocol).; Table S2: Sequential hematologic parameters on the day of the 3rd SBRT fraction and Days 1–12 post-SBRT.; Table S3: Sequential serum biochemistry results from the day of the 3rd SBRT fraction to Day 4 post-SBRT.

## Abbreviations

The following abbreviations are used in this manuscript:

SBRT	Stereotactic body radiotherapy
SRS	Stereotactic radiosurgery
VMAT	Volumetric-modulated arc therapy
GTV	Gross tumor volume
CTV	Clinical target volume
PTV	Planning target volume
OAR	Organs at risk
CT	Computed tomography
HU	Hounsfield unit
FNA	Fine-needle aspiration
PT	Prothrombin time
aPTT	activated partial thromboplastin time
TLS	Tumor lysis syndrome
