# Supplementary material for: Suspected Tumor-Related Hemorrhage as a Rare Complication of Stereotactic Body Radiotherapy in a Dog with Cranial Mediastinal Mass: A Case Report
Source: Vet Sci. 2025 Oct 13;12(10):982. doi: 10.3390/vetsci12100982 (PMC12567663; doi:10.3390/vetsci12100982)
Supplement: Supplementary file 1 [file vetsci-12-00982-s001.zip › vetsci-3880685-supplementary.pdf]

Appendix A

Table S1. Organ-at-risk (OAR) dose constraints applied (3-fraction SBRT protocol).

OAR	Constraint Metric	Applied Threshold (3Fr)
Lung	Dmean	< 6 Gy
	V20Gy	< 10%
Heart	Dmax	< 30 Gy
	D15cc	< 24 Gy
Great vessels	Dmax	< 45 Gy
	D10cc	< 39 Gy
Trachea	Dmax	< 30 Gy
	D4cc	< 15 Gy
Esophagus	Dmax	< 25.2 Gy
	D5cc	< 17.7 Gy
Spinal cord	Dmax	< 21.9 Gy
	D0.35cc	< 18 Gy

Table S2. Sequential hematologic parameters on the day of the 3rd SBRT fraction and Days 1–12 post-SBRT.

Parameter	Reference range	SBRT 3rd fraction	Day 1 post-SBRT	Day 2 post-SBRT	Day 3 post-SBRT	Day 4 post-SBRT	Day 5 post-SBRT	Day 7 post-SBRT	Day 12 post-SBRT
MCV (fL)	60-74	71.9	71.3	71.7	70	70.1	70.1	72.4	74
MCH (pg)	19.5-24.5	23.8	22.2	22.2	22.5	22.9	23	23.2	23.5
MCHC (g/dL)	31-36	33.1	31.2	30.9	32.1	32.6	32.8	32	31.7
RDW (%)	12-18%	13.3	13	12.7	13.1	14.7	16.3	19.1	17.9
Reticulocyte count (10^9/L)	8.4-129.3	137.3	91.1	70.8	113	88.2	178.4	391.3	365.6
Reticulocyte percentage (%)	0.1-2	1.75	2.25	2.05	2.99	2.21	4.07	7.4	6.84
PLT (10^3/μL)	200-500	268	124	82	60	101	128	180	244

Table S3. Sequential serum biochemistry results from the day of the 3rd SBRT fraction to Day 4 post-SBRT.

Parameter	Reference range	SBRT 3rd Fraction	Day 1 post-SBRT	Day 2 post-SBRT	Day 3 post-SBRT	Day 4 post-SBRT
BUN (mg/dL)	5-30	10.8	25.6	13.1	5	10.3
Creatinine (mg/dL)	0.5-1.5	0.8	1.0	0.8	0.5	0.8
Sodium (mmol/L)	139-151	148	143	146	145	146
Potassium (mmol/L)	3.6-5.3	3.9	4.2	4.1	3.7	3.6
Chloride (mmol/L)	107-122	118	119	122	116	117
Calcium (mmol/L)	1.16-1.47	1.34	1.31	1.38	1.32	1.38
Inorganic phosphorus (mg/dL)	2.4-6.4	2.3	5.3	3.4	2.0	1.9
CRP (mg/L)	0-10	7.1	105.5	50.5	24.5	11.8
